# An elevated triglyceride-glucose index predicts adverse outcomes and interacts with the treatment strategy in patients with three-vessel disease

**DOI:** 10.1186/s12933-023-02063-4

**Published:** 2023-12-06

**Authors:** Yu Zhang, Ce Zhang, Lin Jiang, Lianjun Xu, Jian Tian, Xueyan Zhao, Dong Wang, Yin Zhang, Kai Sun, Channa Zhang, Bo Xu, Wei Zhao, Rutai Hui, Runlin Gao, Jizheng Wang, Xinxing Feng, Jinqing Yuan, Lei Song

**Affiliations:** 1 State Key Laboratory of Cardiovascular Disease, Fuwai Hospital, National Center for Cardiovascular Diseases, Chinese Academy of Medical Sciences and Peking Union Medical College, 167, Beilishilu, Xicheng District, Beijing, 100037 People’s Republic of China; 2 Department of Cardiology, Fuwai Hospital, National Center for Cardiovascular Diseases, Chinese Academy of Medical Sciences and Peking Union Medical College, 167, Beilishilu, Xicheng District, Beijing, 100037 People’s Republic of China; 3 Cardiomyopathy Ward, Fuwai Hospital, National Center for Cardiovascular Diseases, Chinese Academy of Medical Sciences and Peking Union Medical College, 167, Beilishilu, Xicheng District, Beijing, 100037 People’s Republic of China; 4 Information Center, Fuwai Hospital, National Center for Cardiovascular Diseases, Chinese Academy of Medical Sciences and Peking Union Medical College, 167, Beilishilu, Xicheng District, Beijing, 100037 People’s Republic of China; 5 Department of Endocrinology, Fuwai Hospital, National Center for Cardiovascular Diseases, Chinese Academy of Medical Sciences and Peking Union Medical College, 167, Beilishilu, Xicheng District, Beijing, 100037 People’s Republic of China; 6 National Clinical Research Center of Cardiovascular Diseases, Fuwai Hospital, National Center for Cardiovascular Diseases, Chinese Academy of Medical Sciences and Peking Union Medical College, 167, Beilishilu, Xicheng District, Beijing, 100037 People’s Republic of China

**Keywords:** Three-vessel disease, Triglyceride-glucose index, Insulin resistance, Diabetes, Revascularisation strategy

## Abstract

**Background:**

Insulin resistance is a pivotal risk factor for cardiovascular diseases, and the triglyceride-glucose (TyG) index is a well-established surrogate of insulin resistance. This study aimed to investigate the prognostic value of the TyG index and its ability in therapy guidance in patients with three-vessel disease (TVD).

**Methods:**

A total of 8862 patients with TVD with available baseline TyG index data were included in the study. The endpoint was major adverse cardiac events (MACE). All patients received coronary artery bypass grafting (CABG), percutaneous coronary intervention (PCI), or medical therapy (MT) alone reasonably.

**Results:**

An elevated TyG index was defined as the TyG index greater than 9.51. During a median follow-up of 7.5 years, an elevated TyG index was significantly associated with an increased risk of MACE (adjusted hazard ratio 1.161, 95% confidence interval 1.026–1.314, p = 0.018). The elevated TyG index was shown to have a more pronounced predictive value for MACE in patients with diabetes, but failed to predict MACE among those without diabetes, whether they presented with stable angina pectoris (SAP) or acute coronary syndrome (ACS). Meanwhile, the association between an elevated TyG index and MACE was also found in patients with left main involvement. Notably, CABG conferred a significant survival advantage over PCI in patients with a normal TyG index, but was not observed to be superior to PCI in patients with an elevated TyG index unless the patients had both ACS and diabetes. In addition, the benefit was shown to be similar between MT and revascularisation among patients with SAP and an elevated TyG index.

**Conclusions:**

The TyG index is a potential indicator for risk stratification and therapeutic decision-making in patients with TVD.

**Graphical Abstract:**

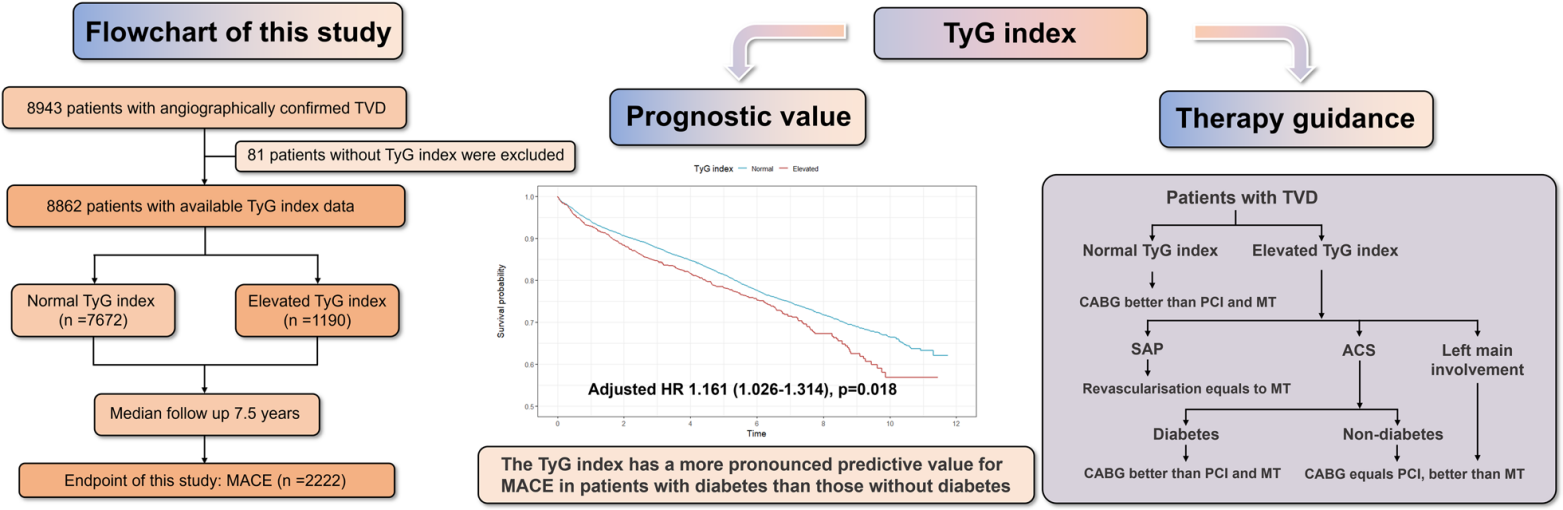

**Supplementary Information:**

The online version contains supplementary material available at 10.1186/s12933-023-02063-4.

## Introduction

Coronary artery disease (CAD) is the most common form of heart disease and remains the leading cause of death [1]. Three-vessel disease (TVD) is an extreme form of CAD that impacts the blood supply of the left anterior descending, left circumflex, and right coronary arteries and is estimated to occur in 30% of all patients with CAD [2]. Patients with TVD have a substantial risk of adverse events [3]. Therefore, timely identification of patients at high risk is essential to improve their prognosis and guide therapy.

Insulin resistance refers to a pathological state in which insulin-sensitive organs become resistant to insulin and reflects disruption of metabolic homeostasis [4, 5]. Insulin resistance is not only the core defect in type 2 diabetes but also one of the most important factors exacerbating vascular lesions in patients with CAD [6]. However, the classical methods used for measurement of insulin resistance (i.e., euglycemic insulin clamp and intravenous glucose tolerance testing) cannot be widely applied in the clinical setting owing to their high cost and invasiveness [7].

An emerging indicator known as the triglyceride-glucose (TyG) index, which is developed from triglycerides and glucose, is a simple and reliable surrogate for insulin resistance and has been shown to perform better than the homeostasis model assessment of insulin resistance [8, 9]. Previous studies have demonstrated the predictive value of the TyG index in patients with stable CAD [10, 11], those with acute coronary syndrome [12–15], and those with myocardial infarction and nonobstructive coronary arteries [16] but not in patients with TVD. A recent study found that an elevated TyG index was associated with a higher risk of multi-vessel involvement in CAD [17], suggesting a distinct role of the TyG index in TVD.

The aim of this study was to investigate the prognostic value of the TyG index and its interaction with the treatment strategy in a large cohort of patients with TVD.

## Methods

### Design

**Fig. 1:**
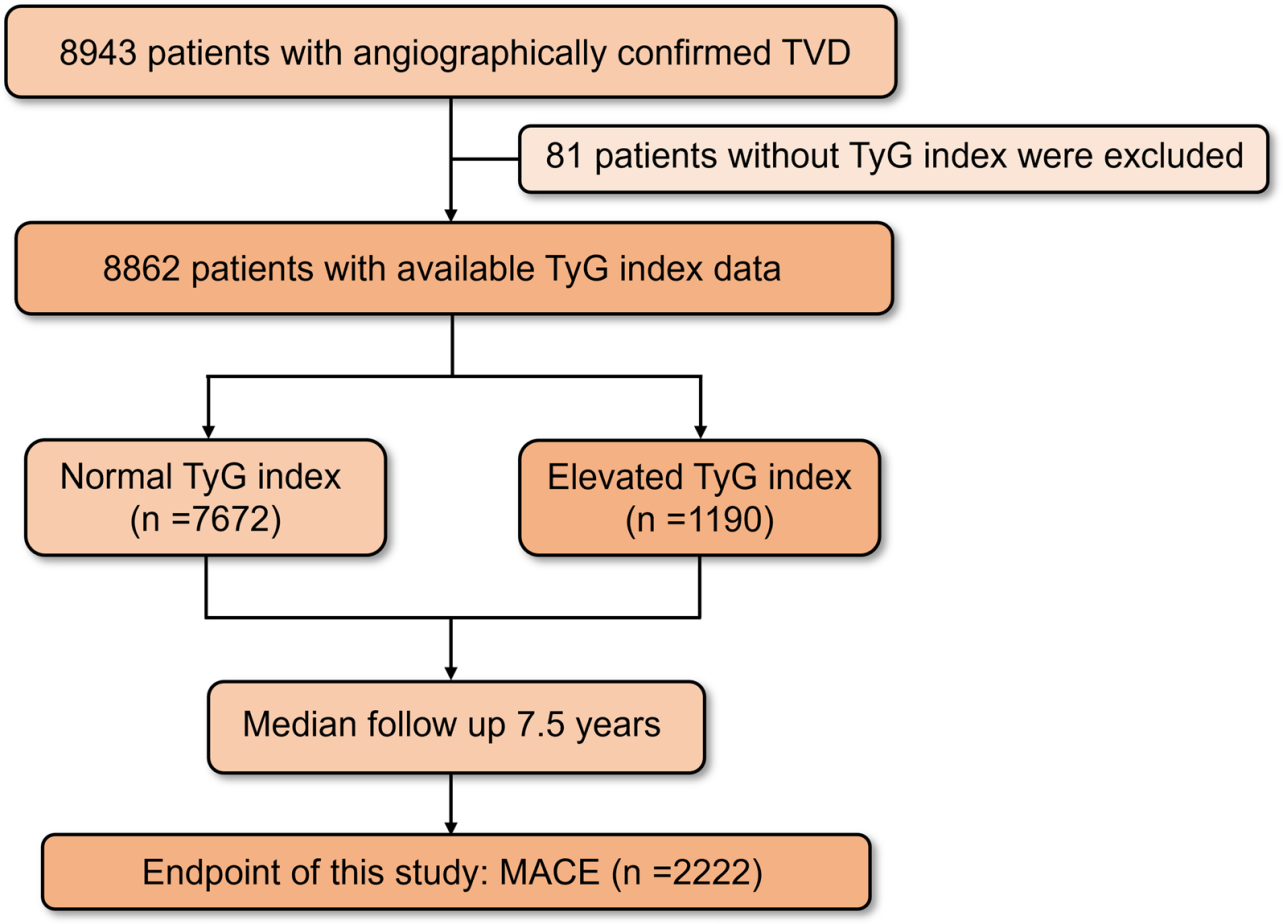
Flowchart of this study. MACE, major adverse cardiac events; TVD, three-vessel disease; TyG index, triglyceride-glucose index

A total of 8943 consecutive patients with TVD were prospectively enrolled at Fuwai Hospital, Chinese Academy of Medical Sciences in Beijing, China, between 2004 and 2011. TVD was defined as angiographically confirmed stenosis of ≥ 50% in all three main epicardial coronary arteries (left anterior descending, left circumflex, and right coronary) with or without involvement of the left main artery. Finally, 8862 patients with available triglyceride and fasting plasma glucose measurements were included in the study. The flowchart of this study is shown in Fig. 1.

All patients underwent a detailed clinical examination and received coronary artery bypass grafting (CABG), percutaneous coronary intervention (PCI), or medical therapy (MT) alone according to current practice guidelines, the judgement of the heart team, and patient preferences. Patients were followed up annually until 2016 by telephone interview, follow-up letter, or clinic visit. Investigators underwent training and a blinded questionnaire was completed to obtain high-quality data.

The study was approved by the Ethics Committee of Fuwai Hospital and performed in accordance with the principles of the Declaration of Helsinki. Informed consent was obtained from all study participants.

### Measurement and calculation of TyG index

Fasting blood samples were collected from the enrolled patients within 24 h after admission. Fasting plasma glucose and triglyceride concentrations were measured using standard biochemical techniques in the core laboratory at Fuwai Hospital. The TyG index was calculated as follows: ln (triglycerides [mg/dL] × fasting plasma glucose [mg/dL]/2).

### Determination of the cut point for the TyG index

The cut point for the TyG index was calculated using the “surv_cutpoint” function (survminer package, Version 0.4.9 in R). The TyG value that corresponded to the most significant relation (the maximal log-rank statistic) with the primary endpoint was thought to be the optimal cut point (Additional file 1: Fig. S1).

### Study endpoint

The endpoint of this study was major adverse cardiac events (MACE), which is a composite of cardiac death, myocardial infarction, stroke, and urgent revascularisation.

### Statistical analysis

Continuous data are shown as the mean ± standard deviation or as the median [interquartile range (IQR)]. Categorical variables are summarised as the percentage. The Student’s *t*-test was used to compare continuous variables between groups, and the chi-squared test was used to compare categorical variables. The statistical analysis was performed using R Version 4.0.2 (R Core Team, Vienna, Austria). A two-sided p-value of < 0.05 was considered statistically significant.

Survival curves were conducted by Kaplan–Meier method and compared using the log-rank test. Cox proportional hazard regression models were used to evaluate the associations between the TyG index and outcomes with calculation of hazard ratio (HRs) and 95% confidence interval (CIs). The multivariable Cox models were adjusted for age, sex, body mass index, previous myocardial infarction, family history of CAD, previous stroke, hypertension, diabetes, hyperlipidaemia, chronic obstructive pulmonary disease, peripheral artery disease, chronic kidney disease, smoking, clinical presentation (acute coronary syndrome [ACS] or stable angina pectoris [SAP]), left ventricular ejection fraction, creatinine clearance, left main involvement, SYNTAX score, and treatment strategy (CABG, PCI, or MT).

### Data and resource availability

The dataset analyzed in the current study are available from the corresponding author upon reasonable request.

## Results

### Patient characteristics at baseline

The mean age of the 8862 study participants was 61.0 ± 10.0 years, and 79.7% were men. The optimal cut point for the TyG index in patients with TVD was identified to be 9.51 (see Methods and Additional file 1: Fig. S1). Levels above this cutoff value were considered elevated. As shown in Table 1, an elevated TyG index was observed in 1190 (13.4%) of all patients and found to be associated with younger age, female sex, and a higher body mass index. The incidence of comorbidities, including diabetes, hypertension, hyperlipidaemia, and chronic kidney disease, was higher in patients with an elevated TyG index than in those with a normal TyG index. Furthermore, patients with an elevated TyG index were more prone to ACS and more likely to have a family history of CAD.

**Table 1:** Baseline characteristics of the study population

	Normal TyG index (≤ 9.51)	Elevated TyG index (> 9.51)	P-value
	n = 7672	n = 1190	
Age (years)	61.4 ± 10.0	59.0 ± 9.8	< 0.001
Male	6227 (81.2)	838 (70.4)	< 0.001
BMI (kg/m^2^)	25.8 ± 3.1	26.3 ± 2.9	< 0.001
Family history of CAD	2147 (28.0)	400 (33.6)	< 0.001
Previous myocardial infarction	2772 (36.1)	381 (32.0)	0.006
Previous stroke	768 (10.0)	113 (9.5)	0.617
Hypertension	5157 (67.2)	835 (70.2)	0.047
Diabetes	2315 (30.2)	779 (65.5)	< 0.001
Hyperlipidaemia	4142 (54.0)	815 (68.5)	< 0.001
COPD	93 (1.2)	7 (0.6)	0.080
PAD	590 (7.7)	99 (8.3)	0.487
CKD	56 (0.7)	17 (1.4)	0.021
Current/former smoker	4306 (56.1)	629 (52.9)	0.037
Clinical presentation			
SAP	3070 (40.0)	430 (36.1)	0.012
ACS	4602 (60.0)	760 (63.9)	0.012
LVEF (%)	58.5 ± 9.3	58.3 ± 9.4	0.474
CCr (mL/min)	84.8 ± 26.5	89.8 ± 28.7	< 0.001
TyG index	8.76 ± 0.39	9.92 ± 0.42	< 0.001
Left main involvement	1827 (23.8)	236 (19.8)	0.003
SYNTAX score			
≤ 22	3027 (39.5)	497 (41.8)	0.317
23–32	2871 (37.4)	429 (36.1)	
≥ 33	1774 (23.1)	264 (22.2)	
Treatment strategy			
CABG	2338 (30.5)	338 (28.4)	0.347
PCI	3269 (42.6)	520 (43.7)	
MT	2065 (26.9)	332 (27.9)	
Medication at discharge			
Aspirin	7327 (95.5)	1149 (96.6)	0.115
Clopidogrel	3985 (51.9)	645 (54.2)	0.155
ACEI	2796 (36.4)	484 (40.7)	0.005
ARB	1151 (15.0)	196 (16.5)	0.204
Beta-blocker	6713 (87.5)	1075 (90.3)	0.006
CCB	2764 (36.0)	432 (36.3)	0.879
Statin	5110 (66.6)	815 (68.5)	0.211

Values are presented as mean ± standard deviation or number (%)

ACEI, angiotensin-converting enzyme inhibitors; ACS, acute coronary syndrome; ARB, angiotensin II receptor blockers; BMI, body mass index; CABG, coronary artery bypass grafting; CAD, coronary artery disease; CCB, calcium channel blocker; CCr, creatinine clearance; CKD, chronic kidney disease; COPD, chronic obstructive pulmonary disease; LVEF, left ventricular ejection fraction; MT, medical therapy; PAD, peripheral artery disease; PCI, percutaneous coronary intervention; SAP, stable angina pectoris; TyG index, triglyceride-glucose Index

### Associations between TyG index and outcomes

The median follow-up duration was 7.5 years [IQR 5.9, 9.1]. During follow-up, 2222 patients (25.1%) experienced MACE.

**Fig. 2:**
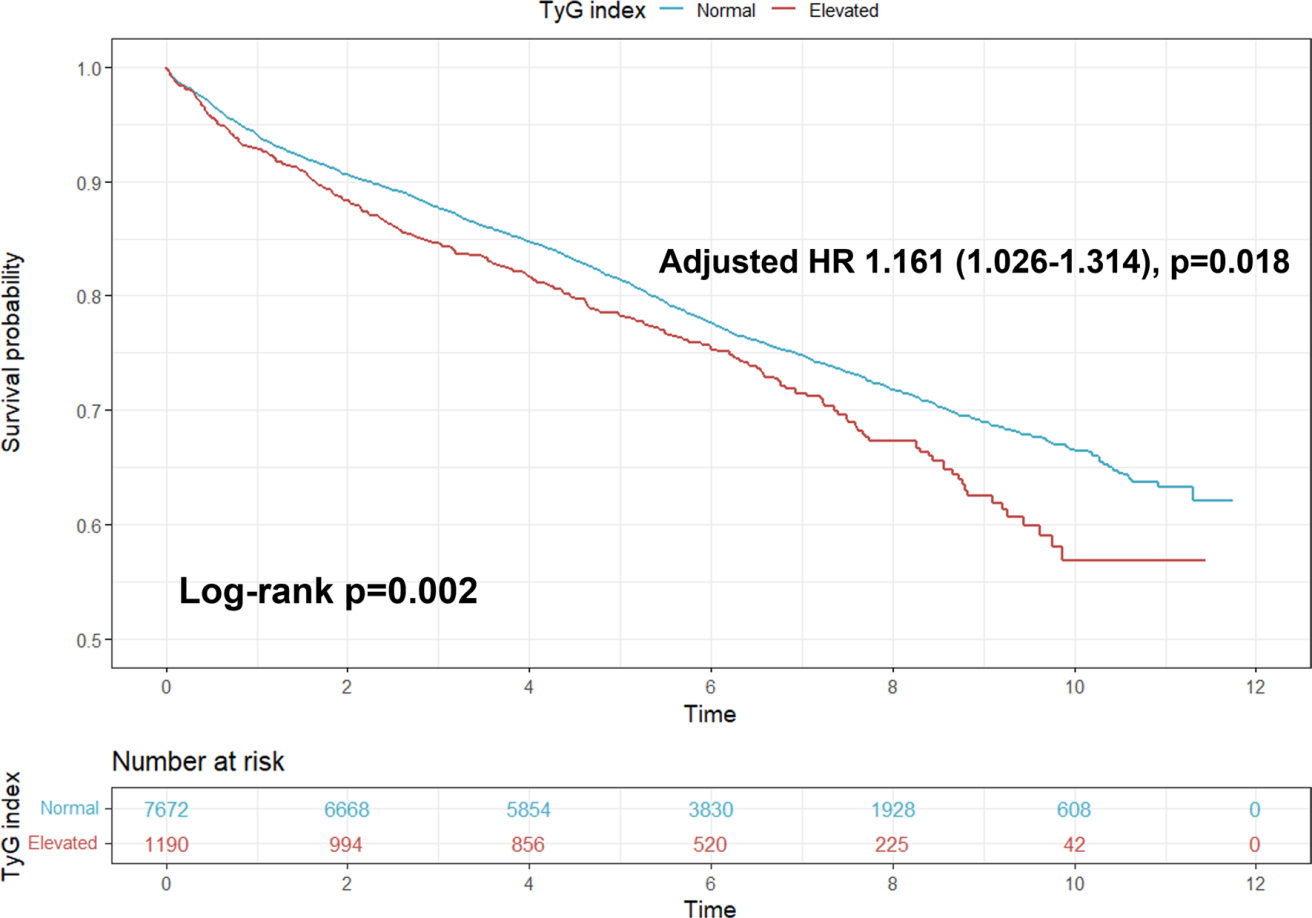
Survival curves for MACE according to TyG index in TVD patients. HR, hazard ratio; TVD, three-vessel disease; TyG index, triglyceride-glucose index

Kaplan–Meier analysis showed that an elevated TyG index was associated with a higher incidence of MACE (p = 0.002, log-rank test) (Fig. 2). Univariable and multivariable Cox analyses showed that an elevated TyG index was significantly associated with an increased risk of MACE (crude HR 1.200, 95% CI 1.067–1.350, p = 0.002; adjusted HR 1.161, 95% CI 1.026–1.314, p = 0.018) (Fig. 2 and Additional file 2: Table S1). Further analysis indicated that there was an additional predictive value of the TyG index over diabetes (Additional file 3: Table S2).

The prognostic impact of the TyG index varies in patients with different disease phenotypes (Additional file 4: Table S3). A subgroup analysis was performed according to the clinical presentations (SAP or ACS) and the presence or absence of diabetes. The results showed that an elevated TyG index increased the risk of MACE only in diabetic patients rather than non-diabetic patients, whether they presented with SAP or ACS. Additionally, the association between an elevated TyG index and MACE was also found in patients with TVD plus left main involvement.

### Interaction between the TyG index and the treatment strategy

**Fig. 3:**
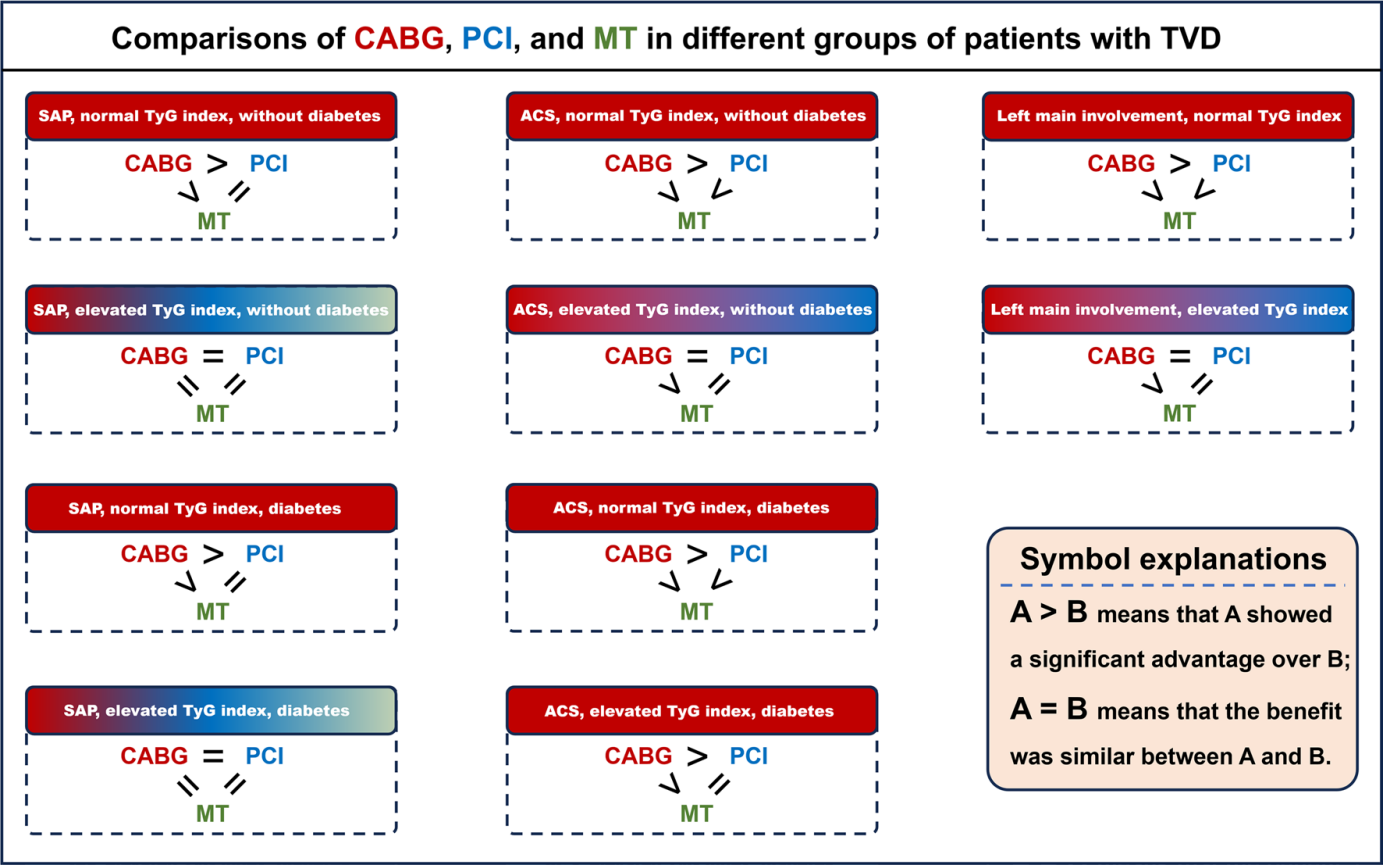
The benefit of three treatment strategies in TVD patients with different disease phenotypes and different levels of the TyG index. A > B means that A showed a significant advantage over B; A = B means that the benefit was similar between A and B. ACS, acute coronary syndrome; CABG, coronary artery bypass grafting; MACE, major adverse cardiac events; MT, medical therapy; PCI, percutaneous coronary intervention; SAP, stable angina pectoris; TVD, three-vessel disease; TyG index, triglyceride-glucose index

In this cohort, 2676 (30.2%), 3789 (42.8%), and 2397 (27.0%) patients received CABG, PCI, or MT alone, respectively. To assess the impact of the TyG index on the choice of treatment strategy selection for patients with TVD, we compared the benefits of three treatment strategies in different disease phenotypes with different levels of the TyG index (Table 2 and Fig. 3).

**Table 2:** Risk of MACE across disease phenotypes, TyG index groups, and treatment strategies

	CABG vs. MT		PCI vs. MT		CABG vs. PCI	
	Adjusted HR (95% CI)	Adjusted P-value	Adjusted HR (95% CI)	Adjusted P-value	Adjusted HR (95% CI)	Adjusted P-value
Grouped by clinical presentations and diabetes*						
Patients with SAP and a normal TyG index, without diabetes	0.443 (0.344–0.570)	< 0.001	0.827 (0.661–1.035)	0.097	0.536 (0.417–0.689)	< 0.001
Patients with SAP and an elevated TyG index, without diabetes	0.776 (0.315–1.910)	0.581	1.136 (0.442–2.921)	0.791	0.683 (0.272–1.714)	0.416
Patients with SAP, diabetes and a normal TyG index	0.568 (0.401–0.804)	0.001	1.049 (0.773–1.425)	0.759	0.541 (0.386–0.759)	< 0.001
Patients with SAP, diabetes and an elevated TyG index	0.541 (0.288–1.018)	0.057	0.874 (0.500–1.527)	0.635	0.620 (0.335–1.146)	0.127
Patients with ACS and a normal TyG index, without diabetes	0.510 (0.417–0.624)	< 0.001	0.790 (0.668–0.933)	0.006	0.646 (0.528–0.790)	< 0.001
Patients with ACS and an elevated TyG index, without diabetes	0.467 (0.219–0.997)	0.049	0.720 (0.375–1.383)	0.324	0.649 (0.303–1.390)	0.265
Patients with ACS, diabetes and a normal TyG index	0.391 (0.290–0.528)	< 0.001	0.758 (0.598–0.960)	0.022	0.516 (0.379–0.703)	< 0.001
Patients with ACS, diabetes and an elevated TyG index	0.309 (0.179–0.533)	< 0.001	0.743 (0.519–1.065)	0.106	0.416 (0.239–0.722)	0.002
Grouped by left main involvement^#^						
Patients with left main involvement and a normal TyG index	0.529 (0.427–0.656)	< 0.001	0.739 (0.572–0.956)	0.021	0.716 (0.551–0.930)	0.012
Patients with left main involvement and an elevated TyG index	0.457 (0.255–0.821)	0.009	0.653 (0.343–1.244)	0.195	0.700 (0.345–1.421)	0.324

*Adjusted for age, sex, body mass index, family history of coronary artery disease, previous myocardial infarction, previous stroke, hypertension, hyperlipidaemia, chronic obstructive pulmonary disease, peripheral artery disease, chronic kidney disease, current/former smoker, left ventricular ejection fraction, creatinine clearance, left main involvement and SYNTAX score. ^#^Adjusted for age, sex, body mass index, family history of coronary artery disease, previous myocardial infarction, previous stroke, hypertension, diabetes, hyperlipidaemia, chronic obstructive pulmonary disease, peripheral artery disease, chronic kidney disease, current/former smoker, acute coronary syndrome, left ventricular ejection fraction, creatinine clearance, and SYNTAX score

ACS, acute coronary syndrome; CABG, coronary artery bypass grafting; CI, confidence interval; HR, hazard ratio; MACE, major adverse cardiac events; MT, medical therapy; PCI, percutaneous coronary intervention; SAP, stable angina pectoris; TyG index, triglyceride-glucose Index

In the group of patients with SAP and a normal TyG index, the risk of MACE after PCI and MT was comparable, and CABG was associated with a lower risk of MACE than PCI and MT; while among patients with SAP and an elevated TyG index, the risk of MACE was similar among three treatment strategies. These findings were regardless of the presence or absence of diabetes.

In the group of patients with ACS and a normal TyG index, both revascularisation strategies were at much lower risk of MACE than MT, and CABG was further associated with a lower risk of MACE than PCI, regardless of the presence or absence of diabetes. When the TyG index was elevated in patients with ACS, the risk of MACE after CABG was similar to that after PCI in those without diabetes; while CABG was shown to be associated with a lower risk of MACE than PCI in those with diabetes.

Moreover, in the group of patients with left main involvement, the risk of MACE was significantly lower after CABG than after PCI among patients with a normal TyG index; while among patients with an elevated TyG index, the risk of MACE after CABG was similar to that after PCI.

In summary, when comparing two revascularisation strategies, patients with a normal TyG index had a greater benefit from CABG than PCI; whereas in the presence of an elevated TyG index, the risk of MACE after CABG and PCI were similar, unless the patients had both ACS and diabetes. MT showed a similar benefit as revascularisation among patients with SAP and an elevated TyG index.

## Discussion

In this large cohort of patients with TVD, we showed a significant association of an elevated TyG index with an increased risk of MACE. For distinct phenotype groups, an elevated TyG index had a predictive role for MACE in patients with diabetes and not in those without diabetes, regardless of the clinical presentations (SAP or ACS); the predictive role of the TyG index was also observed in patients with TVD plus left main involvement. CABG afforded a significant advantage over PCI and MT in terms of MACE among patients with a normal TyG index. However, CABG was not observed to be superior to PCI in patients with an elevated TyG index unless the patients had both ACS and diabetes. In addition, the benefit was shown to be similar between MT and revascularisation among patients with SAP and an elevated TyG index.

Insulin resistance can promote both atherogenesis and progression of plaque, thus correlates well with an increased risk of atherothrombotic cardiovascular disease [18, 19]. As a reliable indicator of insulin resistance, the TyG index has been demonstrated by many studies to have a positive relationship with the incidence of CAD [20, 21]. An elevated TyG index indicates severe systemic disruption of lipid metabolism and glucose, which is associated with impairment of coronary endothelial and smooth muscle cells as well as escalation of the risk of CAD [22]. More importantly, the TyG index has been identified to have a prognostic role in several types of CAD, including stable CAD [10, 11], acute coronary syndrome [12–15], and myocardial infarction with nonobstructive coronary arteries [16]. However, the prognostic usefulness of the TyG index remains unclear in patients with TVD. In the present study, we found that the TyG index had fair prognostic ability in patients with this complex and severe type of CAD.

Diabetes is one of the most significant risk factors for CAD, and insulin resistance promotes development of type 2 diabetes [23]. Based on the high degree of causality between insulin resistance and diabetes, we performed a subgroup analysis according to the presence of concomitant diabetes in patients with TVD and found that an elevated TyG index was associated with an increased risk of MACE in the diabetic subgroup but not in the non-diabetic subgroup, whether the patients presented SAP or ACS. This result might be explained by the overall low insulin resistance in patients without diabetes and is consistent with previous studies showing that the TyG index has a stronger predictive value in patients with CAD and diabetes than in their non-diabetic counterparts [11, 13, 15, 16, 24]. Moreover, an elevated TyG index also conferred valid predictive value on patients with left main involvement that represents a subgroup at a high risk.

The current guidelines recommend CABG as the preferred treatment strategy for multivessel CAD owing to its advantages in management of complex lesions and less need for repeat revascularisation [25, 26] However, our results suggested that the benefit of three treatment strategies (CABG, PCI, and MT) was closely related to the levels of TyG index and disease phenotypes in patients with TVD. We found that CABG was the optimal treatment strategy among patients with a normal TyG index, regardless of the clinical presentations, the presence or absence of diabetes, and left main involvement; while the survival benefit of CABG over PCI disappeared when patients had an elevated TyG index. This may be because the risk of symptomatic graft failure after CABG is higher in patients with an elevated TyG index than in those with a normal TyG index [27], which worsens both short-term and long-term outcomes [28]. Another interpretation of this result is that perioperative and postoperative stress responses deteriorate the impairment of insulin signaling in patients with insulin resistance, who already have severe metabolic syndrome [29, 30]. It should be noted that CABG remained the optimal choice once TVD patients with an elevated TyG index had both ACS and diabetes. This was probably because PCI and MT were hard to manage the extremely severe in situ vascular injury in such patients, and only the revascularisation on relatively healthy grafts could improve the prognosis [31]. In addition, another important finding of our study was the comparable prognosis between MT and revascularization among patients with SAP and an elevated TyG index, suggesting that revascularization might not be superior to MT alone in such patients.

In conclusion, we have demonstrated that an elevated TyG index is a strong risk factor for a poor prognosis in patients with TVD, especially those with comorbid diabetes. Moreover, we found that CABG is the preferred treatment in patients with TVD and a normal TyG index because of the lower risk of MACE in comparison with PCI and MT, as recommended in the guidelines. However, the optimal treatment strategy in patients with TVD and an elevated TyG index should be re-evaluated unless they have both ACS and diabetes.

This study has some limitations. First, its participants were recruited from a single centre, which might limit the generalisability of its findings. Second, the study had an observational design, which means that the possibility of several types of bias cannot be excluded, even after strict adjustment for confounders. Therefore, our findings require confirmation in randomised controlled clinical trials in the future. Third, the validity of the TyG index on admission is limited in the acute phase of ACS, because it might not represent the real degree of insulin resistance in the stable phase and during the follow-up. Further analysis of the trajectory of the TyG index might help to address this point.

### Supplementary Information

**Additional file 1: Figure S1.** The optimal cut point of TyG index.

**Additional file 2: Table S1.** Details of the univariable and multivariable analysis of the association between TyG index and endpoint.

**Additional file 3: Table S2.** The additional predictive value of the TyG index over diabetes for MACE.

**Additional file 4: Table S3.** Associations between elevated TyG index and MACE in different disease phenotype groups.

## Data Availability

The datasets used/or analyzed during the current study are available from the corresponding author on reasonable request.

## Abbreviations

ACS: Acute coronary syndrome
CABG: Coronary artery bypass grafting
CAD: Coronary artery disease
CI: Confidence interval
IQR: Interquartile range
MACE: Major adverse cardiac events
MT: Medical therapy
HR: Hazard ratio
PCI: Percutaneous coronary intervention
SAP: Stable angina pectoris
TVD: Three-vessel disease
TyG index: Triglyceride-glucose index
