# Supplementary figures and images for: An elevated triglyceride-glucose index predicts adverse outcomes and interacts with the treatment strategy in patients with three-vessel disease
Source: Cardiovasc Diabetol. 2023 Dec 6;22:333. doi: 10.1186/s12933-023-02063-4 (PMC10702040; doi:10.1186/s12933-023-02063-4)

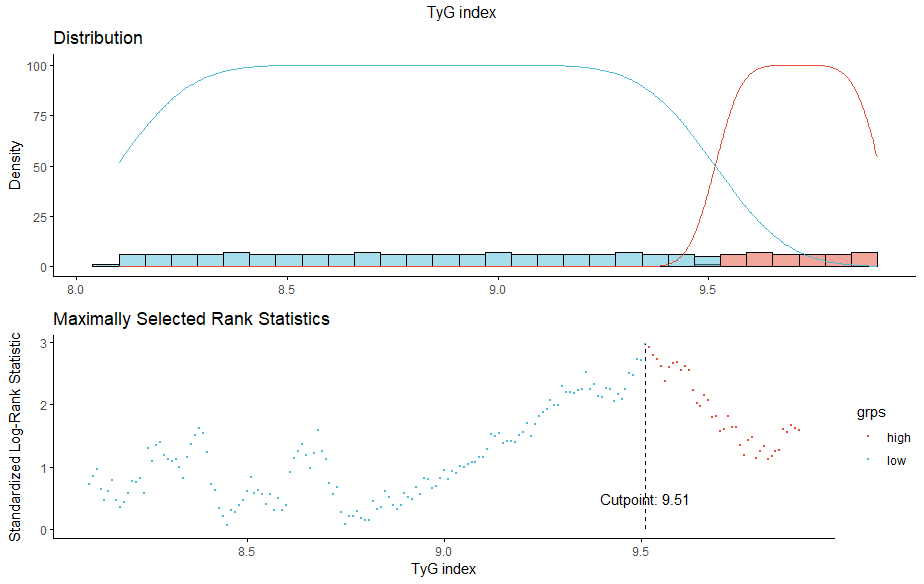

Supplement: Supplementary file 1 — Additional file 1: Figure S1. The optimal cut point of TyG index. [file 12933_2023_2063_MOESM1_ESM.tiff]
